# Epidemiology of Bacteremia in Patients with Hematological Malignancies and Hematopoietic Stem Cell Transplantation and the Impact of Antibiotic Resistance on Mortality: Data from a Multicenter Study in Argentina

**DOI:** 10.3390/pathogens13110933

**Published:** 2024-10-26

**Authors:** Fabián Herrera, Diego Torres, Ana Laborde, Rosana Jordán, Lorena Berruezo, Inés Roccia Rossi, Noelia Mañez, Lucas Tula, María Laura Pereyra, Andrea Nenna, Patricia Costantini, José Benso, María Luz González Ibañez, María José Eusebio, Nadia Baldoni, Laura Alicia Barcán, Sandra Lambert, Martín Luck, Fernando Pasterán, Alejandra Corso, Melina Rapoport, Federico Nicola, María Cristina García Damiano, Renata Monge, Ruth Carbone, Mariana Reynaldi, Graciela Greco, Miriam Blanco, María Laura Chaves, Marcelo Bronzi, Alberto Carena

**Affiliations:** 1 Infectious Diseases Section, Internal Medicine Department, Centro de Educación Médica e Investigaciones Clínicas, (CEMIC), Buenos Aires C1431, Argentina; diegots23@hotmail.com (D.T.); alberto.carena390@gmail.com (A.C.); 2 Infectious Diseases Service, Fundación para Combatir la Leucemia (FUNDALEU), Buenos Aires C1114, Argentina; anarlaborde@gmail.com (A.L.); lgonzalez@fundaleu.org.ar (M.L.G.I.); 3 Infectious Diseases Service, Hospital Británico de Buenos Aires, Buenos Aires C1280, Argentina; rosanajordan61@gmail.com (R.J.); joule_cba@hotmail.com (M.J.E.); 4 Infectious Diseases Service, Hospital Interzonal General de Agudos (HIGA) Dr. Rodolfo Rossi, La Plata B1902, Argentina; lvberruezo@gmail.com (L.B.); nadiabaldoni@hotmail.com (N.B.); 5 Infectious Diseases Service, Hospital Interzonal General de Agudos (HIGA) Gral. San Martín, La Plata B1900, Argentina; ainesines@hotmail.com; 6 Infectious Diseases Section, Internal Medicine Department, Hospital Italiano de Buenos Aires, Buenos Aires C1199, Argentina; noelia.manez@hospitalitaliano.org.ar (N.M.); laura.barcan@hospitalitaliano.org.ar (L.A.B.); 7 Infectious Diseases Service, Hospital El Cruce, Buenos Aires B1888, Argentina; lft1969@yahoo.com.ar (L.T.); sandralambert2000@yahoo.com.ar (S.L.); 8 Infectious Diseases Service, Hospital Universitario Austral, Buenos Aires B1629, Argentina; laurapereyra@yahoo.com.ar; 9 Infectious Diseases Service, Hospital Municipal de Oncología Marie Curie, Buenos Aires C1405, Argentina; andrea_nenna@yahoo.com.ar; 10 Infectious Diseases Service, Instituto de Oncología Ángel H. Roffo, Buenos Aires C1417, Argentina; patricostantini@gmail.com (P.C.); martinlu100@hotmail.com (M.L.); 11 Infectious Diseases Section, Internal Medicine Department, Hospital Italiano de San Justo, Buenos Aires C1198, Argentina; jose.benso@hospitalitaliano.org.ar; 12 Antimicrobial Service, INEI-ANLIS Dr. Carlos Malbrán, Buenos Aires C1282, Argentina; fpasteran@gmail.com (F.P.); corsoalejandra@gmail.com (A.C.); rapoport@anlis.gov.ar (M.R.); 13 Microbiology Laboratory, Centro de Educación Médica e Investigaciones Clínicas (CEMIC), Buenos Aires C1431, Argentina; fgnicola@hotmail.com; 14 Microbiology Laboratory, Fundación para Combatir la Leucemia (FUNDALEU), Buenos Aires C1114, Argentina; mcgdamiano@gmail.com; 15 Microbiology Service, Hospital Británico de Buenos Aires, Buenos Aires C1280, Argentina; rlmonge@hbritanico.com.ar; 16 Bacteriology Laboratory, Hospital Interzonal General de Agudos (HIGA) Prof. Dr. Rodolfo Rossi de La Plata, Buenos Aires B1902, Argentina; ruthjcarbone@gmail.com; 17 Microbiology Laboratory, Hospital Interzonal General de Agudos (HIGA), Gral. San Martín de La Plata, Buenos Aires B1900, Argentina; marianareynaldi@gmail.com; 18 Bacteriology Laboratory, Hospital Italiano de Buenos Aires, Buenos Aires C1199, Argentina; graciela.greco@hospitalitaliano.org.ar; 19 Microbiology Laboratory, Hospital de Alta Complejidad El Cruce, Buenos Aires B1888, Argentina; miriblanco@yahoo.com; 20 Microbiology Laboratory, Hospital Municipal de Oncología Marie Curie, Buenos Aires C1405, Argentina; marialaurachaves@hotmail.com; 21 Microbiology Laboratory, Instituto de Oncología Ángel H. Roffo, Buenos Aires C1417, Argentina; mabro_bacterio@yahoo.com.ar

**Keywords:** bacteremia, hematological malignancies, epidemiology, resistance, mortality

## Abstract

The epidemiology of bacteremia and the antibiotic resistance profile (ARP) of Gram-negative bacilli (GNB) in hematological malignancies (HM) and hematopoietic stem cell transplant (HSCT) patients may differ according to geographic region. In addition, multidrug-resistant organisms (MDROs) may impact mortality. This is a prospective, observational, and multicenter study. The first episodes of bacteremia in adult patients with HM or HSCT were included. The risk factors for 30-day mortality were identified. One thousand two hundred and seventy-seven episodes were included (HM: 920; HSCT: 357). GNB were isolated in 60.3% of episodes, with Enterobacterales (46.9%) and *P. aeruginosa* (8.5%) being the most frequent. Gram-positive cocci were isolated in 41.9% of episodes, with coagulase-negative staphylococci (19.8%) and *S. aureus* (10.4%) being the most frequent. MDROs were isolated in 40.2% (24.4% GNB). The ARP of GNB in patients with HM vs. HSCT was cefepime: 36.8% vs. 45.7% (*p* = 0.026); piperacillin–tazobactam: 31.05% vs. 45.2% (*p* < 0.0001); carbapenems: 18.9% vs. 27.3% (*p* = 0.012); and aminoglycosides: 9.3% vs. 15.4% (*p* = 0.017), respectively. Overall mortality between patients with HM and HSCT was 17.5% vs. 17.6% (*p* = 0.951), respectively. The risk factors for mortality were relapsed and refractory underlying disease, corticosteroids use, respiratory source, septic shock, and GNB resistant to meropenem, while 7-day clinical response was a protective factor for survival. Bacteremia was frequently caused by GNB, with a large proportion of MDROs and a high level of antibiotic resistance, especially in patients with HSCT. Carbapenem-resistant GNB bacteremia was associated with a significant increase in mortality.

## 1. Introduction

Bacteremia is the most important infectious complication encountered by patients with hematological malignancies (HM) and hematopoietic stem cell transplantation (HSCT), leading to high morbidity and mortality rates [1,2,3]. A large study in neutropenic patients with Gram-negative bacilli (GNB) bacteremia reported an overall and infection-related mortality of 23.3% and 17.1%, respectively [4]. The epidemiology of bacteremia may differ in terms of geographic region or country and can change over time. Two multicenter studies addressed this issue. In Spain, the most frequent etiological agents of GNB bacteremia were *Escherichia coli* and *Pseudomonas aeruginosa*, while in the Andean region they were *Klebsiella pneumoniae* and *E. coli.* [5,6]. Since the last decade, there has been a predominance of Gram-negative bacilli (GNB) as the leading cause and Enterobacterales as the etiological agents most frequently involved, followed by *Pseudomonas aeruginosa* [7,8,9]. More importantly, these bacteria are likely to develop high antibiotic resistance. Nowadays, multidrug-resistant GNB (MDR-GNB) in this population is a major concern worldwide, mainly due to extended-spectrum beta-lactamase (ESBL) Enterobacterales, carbapenemase-producing Enterobacterales, and MDR *P. aeruginosa* [10,11]. Overall, infection-related mortality can be extremely high in bacteremia episodes caused by the last two pathogens, especially in neutropenic patients. A multicenter study conducted in Italy showed that 21-day mortality rate for patients with carbapenem-resistant *K. pneumoniae* was 52.2%. Moreover, 42.2% mortality rate was reported in MDR-*P. aeruginosa* bacteremia [12,13]. The poor outcome was probably due to the high rate of inadequate empirical antibiotic treatment prescribed and the limited therapeutic options for these infections [5,13,14]. Therefore, knowledge of the local epidemiology and antibiotic resistance profile is crucial for a more appropriate approach to these patients.

In this sense, clinical scores and machine learning algorithms have been developed to predict antibiotic resistance phenotypes and the risk of MDR-GNB [4,15,16].

The present study aimed to describe and compare the etiology of bacteremia, resistance mechanisms, and antimicrobial resistance profiles of GNB in patients with HM and HSCT. We further aimed to identify the risk factors for 30-day mortality.

## 2. Materials and Methods

### 2.1. Study Design

A multicenter prospective study was conducted in 11 referral academic centers specialized in the care of oncological and HSCT patients in Argentina. These centers were chosen given their similarity in terms of patient characteristics and medical treatment provided. Three were cancer centers (2 public and 1 private), with 24 to 80 beds. The others were general hospitals (3 public and 5 private), with 15 to 26 beds allocated to HM and HSCT patients. All episodes of initial bacteremia (defined as the first episode of bacteremia experienced during an admission) in adult patients (≥18 years of age) with HM or HSCT, who were managed as inpatients from May 2014 to April 2020, were included. The following inclusion criteria were met: (a) patients presented with an HM treated with chemotherapy or biological agents (six months prior to admission), or they had been receiving steroids (at a dose equal to or higher than prednisone 20 mg daily or equivalent) for at least two weeks prior to admission; or (b) patients with allogeneic HSCT (with graft versus host disease at any time or without this condition in the first two years) or autologous HSCT (in the first year post-transplant). Patients receiving palliative care and those with recurrent bacteremia were excluded from the analysis.

Patients were included in the study at the time of positive blood culture, whether they had started empirical antibiotic therapy (AT) or not, and were then prospectively followed on a daily basis. Data were obtained from direct patient care, electronic and paper medical records, and microbiological records from the laboratory. Clinical, microbiological, treatment, and outcome variables were evaluated and appropriately defined to avoid inconsistencies. Given the prospective design, missing data were not allowed. Empirical AT was prescribed according to the patient’s clinical and epidemiological features, pursuant to each center’s institutional guidelines and IDSA and ECIL recommendations [17,18,19]. The definitive therapy was selected based on the isolated bacteria and their antibiotic resistance profile. Patients were followed for 30 days after the episode (by direct patient care in hospitalized cases, or by phone calls in the case of discharged patients).

### 2.2. Definitions

Bacteremia was classified as nosocomial, healthcare-associated, or community-acquired according to Friedman et al. [20]. Breakthrough bacteremia was defined as an episode of continuous or new-onset bacteremia in a patient receiving appropriate antibiotics for the microorganism recovered from blood cultures.

In order to determine the clinical source of bacteremia, isolation of the bacterium from the suspected source and/or the associated clinical signs and symptoms were considered. The US CDC criteria were used for the classification [21]. Neutropenic enterocolitis was defined according to Nesher L. et al. [22]

Neutropenia was defined as an absolute neutrophil count < 500 cells/mm^3^. High-risk febrile neutropenia was defined according to the Multinational Association for Supportive Care in Cancer (MASCC) score < 21 and one or more clinical criteria [17,18]. Refractory underlying disease was defined as HM with no response to oncological treatment according to the standard criteria for each disease. Relapse disease was defined as signs and symptoms, images, or molecular evidence of further occurrence of active disease in patients that had achieved complete remission. Septic shock was defined as the need for vasopressors to maintain mean arterial pressure ≥ 65 mmHg and serum lactate level > 18 mg/dL [23]. Infection severity and mortality probability were defined using Pitt and APACHE-II scores.

Empirical AT was considered appropriate provided that it was started after blood cultures were drawn and one or more antibiotics used were active in vitro against the isolated bacteria, with adequate dosing and dose intervals. In patients with ESBL-producing Enterobacterales, empirical or definitive AT with piperacillin–tazobactam or cefepime monotherapy was considered inappropriate [24]. In patients with isolation of any Enterobacterales species, empirical or definitive therapy with tigecycline as monotherapy was deemed inappropriate.

Antibiotic use refers to any antibiotic prescribed for the treatment of bacteremia. Fluoroquinolones prophylaxis was defined as the use of these antibiotics to prevent infections in high-risk neutropenic patients. Clinical response on day 7 of antibiotic therapy was defined as absence of fever for at least four days, source control of bacteremia, absence of hypotension, and clinical resolution of all signs and symptoms of infection.

In case of microbiological, histological, or clinical evidence of active infection, mortality was considered to be related to infection.

### 2.3. Microbiological Studies

Bacteremia was defined as the isolation of pathogenic bacteria in at least one bottle of blood culture (BD BACTEC^TM^ Plus Aerobic/F and Plus Anaerobic/F), analyzed with BD BACTEC (Becton Dickinson, Sparks, MD, USA) or BacTALERT 3D (bioMérieux Inc., Durham, NC, USA), depending on the method available at each center, for a minimum incubation period of five days. Typical skin flora, such as coagulase-negative staphylococci, was considered the cause of bacteremia if two sets of blood cultures were positive for the same species and had an identical antimicrobial profile. MDR Gram-positive cocci included methicillin-resistant *Staphylococcus aureus* or coagulase-negative staphylococci resistant to three or more antibiotics and vancomycin-resistant enterococci. MDR-GNB were defined as GNB resistant to three or more of the following categories of antibiotics: carbapenems, piperacillin–tazobactam, third- and fourth-generation cephalosporins, aztreonam, fluoroquinolones, or aminoglycosides [25,26]. Microbiological identification and susceptibility testing were performed with manual biochemical and microbiological methods, disk diffusion (according to the CLSI recommendations), and/or Etest, VITEK II Compact (bioMérieux, Marcy-l’Étoile, France), PHOENIX 100 BD automated system (Becton Dickinson), VITEK MS (bioMérieux), and MALDI-TOF (BD Bruker Microflex MALDI Biotyper, Bruker Daltonics, Bremen, Germany). ESBL production was determined by disk diffusion method using both ceftazidime and cefotaxime, alone and combined with clavulanic acid. Carbapenemase production was investigated in carbapenem-resistant bacteria using the modified Hodge method, disk synergy tests with a carbapenem disk placed close to the boronic acid disk test for KPC, and the EDTA disk for identification of metallo-β-lactamases. The presence of genes coding for *bla*KPC and *bla*OXA-48 was investigated by monoplex or multiplex polymerase chain reaction (PCR) using specific primers depending on the method available at each center. Multiplex PCR for *bla*VIM, *bla*NDM, *bla*IMP, *bla*KPC, and *bla*OXA-48 was used to investigate isolates at the National Reference Laboratory of Microbiology (ANLIS-Malbrán) [27]. In order to detect colonization with KPC-producing Enterobacterales, rectal swabs were routinely collected and seeded in chromogenic media (CHROMAgar, Paris, France) once a week and in every pre-transplant evaluation in 10 of the 11 centers included in the study. Additionally, a multiplex PCR was performed directly from rectal swabs in 2 centers.

### 2.4. Statistical Analysis

Descriptive statistics were used to characterize the study population. For continuous variables, centrality (median) and dispersion (interquartile range [IQR]) measures were used according to the distribution of variables. Categorical variables were analyzed using absolute frequency and percentage. Groups were compared using U Mann–Whitney test for continuous variables and Fisher exact test or chi-square test for categorical variables. For all tests, a 95% level of statistical significance was used.

To identify the risk factors for 30-day mortality, a multiple logistic regression model was used. Variables with *p* < 0.05 in the univariate analysis were included in the multivariate model. All reported *p-*values are 2-tailed.

For the logistic regression model used to analyze 30-day mortality, we calculated the sample size required to achieve a 5% precision level. The analysis revealed that approximately 982 subjects were needed to provide 80% statistical power with a significance level of 0.05.

Analyses were performed with the SPSS (Statistics for Windows, Version 22.0. Armonk, NY, USA) software packages.

## 3. Results

A total of 1763 episodes of bacteremia were assessed, and 486 were excluded because they failed to meet the eligibility criteria: 298 were solid tumors and 188 were the second or third episode during an admission. The total study population consisted of 1277 episodes of bacteremia (920 in patients with HM and 357 in patients with HSCT). The median age was 52 years (IQR: 37–63), being higher in HM, and 58.1% were male, with a larger rate in HSCT. Although the median Charlson comorbidity score was 2 (IQR: 2–2), in a larger number of HM patients, it was ≥3. The most frequent underlying diseases were acute leukemias, lymphomas, and multiple myeloma, with the former being more common in patients with HM and the latter in patients with HSCT. Among HSCT patients, 155 (43.4%) were allogeneic. While most HM patients had active disease, a high proportion of HSCT patients had the disease in complete or partial remission. Eight hundred and ninety (69.6%) patients received chemotherapy one month prior to their bacteremia episode, which was more common in patients with HM. One hundred and seventy-six (13.7%) patients were treated with anti-lymphocyte drugs or biological agents, and around one-third of patients were treated with high doses of corticosteroids, with no differences between both groups. Nine hundred and twenty-four (72.3%) patients were neutropenic, and most of them were at high risk. Baseline and epidemiological characteristics among patients with HM and HSCT are outlined in Table 1.

### 3.1. Microbiological Characteristics and Antibiotic Resistance Patterns

Regarding the microbiological characteristics, 60.4% of bacteremias were caused by GNB, 41.9% by Gram-positive cocci, and 6.6% were polymicrobial.

**Figure 1:**
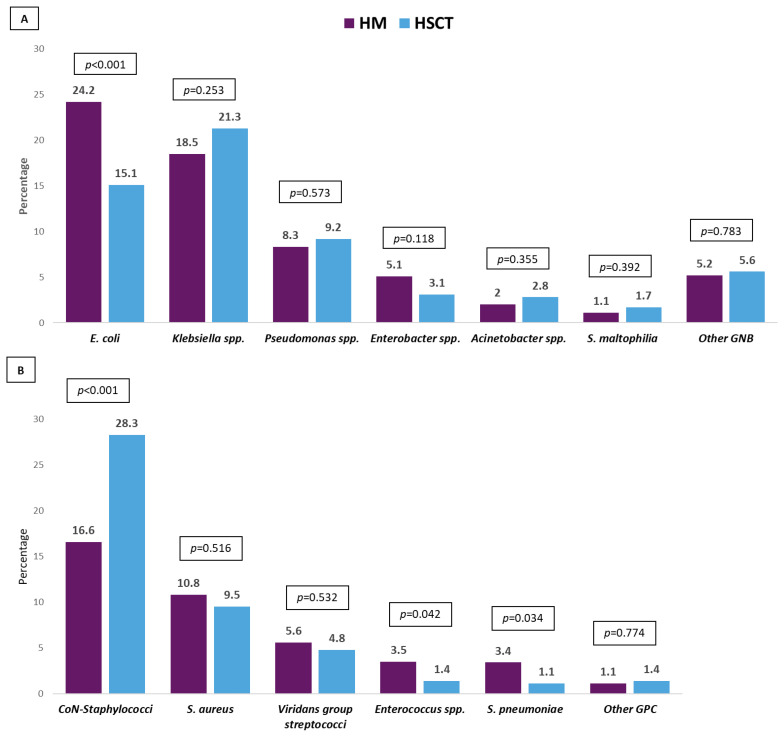
Etiology of bacteremia episodes in patients with hematologic malignancies (HMs) vs. hematopoietic stem cell transplant recipients (HSCT). (**A**): Gram-negative bacilli. (**B**): Gram-positive cocci. Abbreviation: CoN-staphylococci: Coagulase-negative staphylococci. *p*-value obtained by chi-square or Fisher exact test.

**Figure 2:**
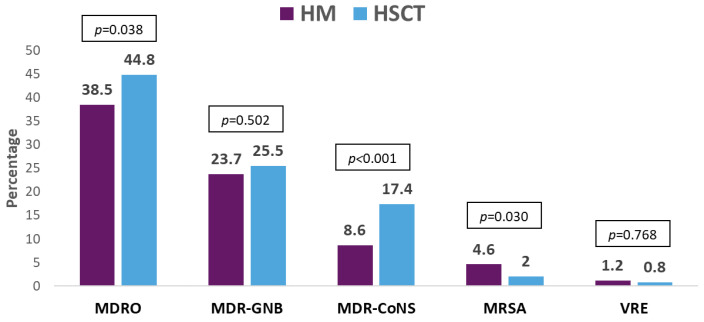
Frequency and type of multidrug-resistant organisms in patients with hematologic malignancies (HMs) vs. hematopoietic stem cell transplant recipients (HSCT). Abbreviation: MDRO, multidrug-resistant organisms; MDR-GNB, multidrug-resistant Gram-negative bacilli; MDR-CoNS, multidrug-resistant coagulase-negative staphylococci; MRSA, methicillin-resistant *Staphylococcus* aureus; VRE, vancomycin-resistant enterococci. *p*-value obtained by chi-square or Fisher exact test.

**Figure 3:**
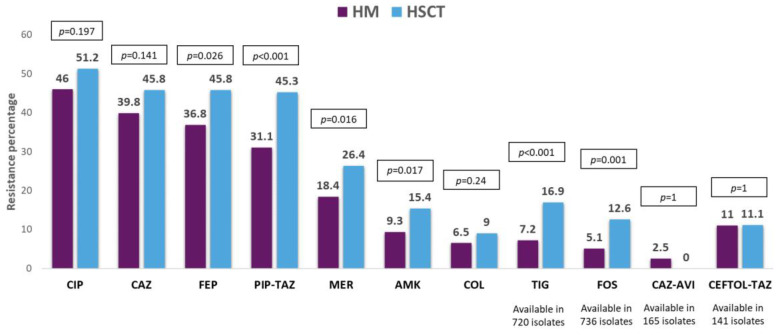
Resistant profiles (resistance percentage) of the Gram-negative bacilli bacteremia in patients with hematologic malignancies (HMs) vs. hematopoietic stem cell transplant (HSCT) recipients. Abbreviation: CIP, ciprofloxacin; CAZ, ceftazidime; FEP, cefepime; PIP-TAZ, piperacillin–tazobactam; MER, meropenem; AMK, amikacin; COL, colistin; TIG, tigecycline; FOS, fosfomycin; CAZ-AVI, ceftazidime–avibactam; CEFTOL-TAZ, ceftolozane–tazobactam. *p*-value obtained by chi-square or Fisher exact test.

**Figure 4:**
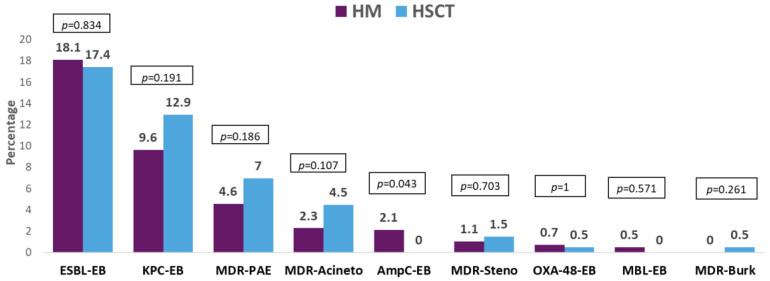
Resistance mechanisms (or phenotype) of the Gram-negative bacilli bacteremia in patients with hematologic neoplasms (HMs) vs. hematopoietic stem cell transplant (HSCT) recipients. Abbreviation: ESBL-EB, extended-spectrum beta-lactamase-producing Enterobacterales; KPC-EB, *Klebsiella pneumoniae* carbapenemase-producing Enterobacterales; MDR-PAE, multidrug-resistant *Pseudomonas aeruginosa*; MDR-Acineto, multidrug-resistant *Acinetobacter* spp; Amp C-EB, Amp C-producing Enterobacterales; MDR-Steno, multidrug-resistant *Stenotrophomonas maltophilia*; OXA-48-like-producing Enterobacterales; MBL-EB, metallo-beta-lactamase-producing Enterobacterales; MDR-Burk, multidrug-resistant *Burkholderia* spp. *p*-value obtained by chi-square or Fisher exact test.

Five hundred and ninety-nine (77.6%) GNB bacteremias were caused by Enterobacterales followed by *P. aeruginosa* (109, 14.1%). *Escherichia coli* was more frequently isolated from patients with HM, while *Klebsiella* spp. was the main isolated bacteria in HSCT. Coagulase-negative staphylococci were isolated in half of Gram-positive bacteremias (being more frequent in patients with HSCT), and *Enterococcus* spp. and viridans group streptococci were isolated in a small proportion of the episodes. MDR organisms (MDROs) were isolated in 514 (40.2%) episodes, being more frequent in HSCT. More than half of these isolates were MDR-GNB. The resistance mechanisms and phenotypes in order of frequency were ESBL-producing Enterobacterales, KPC-producing Enterobacterales, MDR *P. aeruginosa*, and MDR *Acinetobacter* spp. The resistance profile of the antibiotic most commonly used in this patient population was cefepime (39.2%), piperacillin–tazobactam (34.8%), meropenem (20.5%), and amikacin (10.9%). For all of them, resistance was higher in isolates from patients with HSCT. Resistance to the antibiotics commonly used for the treatment of MDR-GNB was colistin (7.1%), tigecycline (9.7%), and fosfomycin (7.1%), with the last two being high in carbapenem-resistant isolates from HSCT patients. Meanwhile, resistance to ceftolozane–tazobactam and ceftazidime–avibactam was low in the isolates tested against these antibiotics. The etiological profile of bacteremias, frequency of MDROs, resistance mechanisms and phenotypes, and antibiotic resistance profiles are described in Figure 1, Figure 2, Figure 3 and Figure 4.

### 3.2. Clinical Characteristics, Treatment, and Outcomes

Nine hundred and sixteen (71.7%) bacteremias had a clinical source, with the most frequent being central-venous catheter, abdomen (all were colitis), and lower respiratory tract. Median APACHE II and Pitt scores were 13 (IQR: 10–17) and 0 (0–2), with no differences between the groups.

One thousand seventy-three (84%) patients received appropriate empirical AT, and the antibiotics most frequently prescribed were piperacillin–tazobactam (41.7%) and carbapenems (40.7%). Four hundred and sixty-three (36.2%) patients, most of them in the HSCT group, received combined empirical AT.

Definitive antibiotic treatment was mainly prescribed as monotherapy (1096, 85.8%), with no differences between groups.

Regarding outcome variables, no differences were observed between patients with HM and HSCT in intensive care unit (ICU) admission, septic shock development, breakthrough bacteremia, 7-day clinical response, 7-day mortality, and 30-day overall and infection-related mortality. Clinical characteristics, treatment, and outcomes are described in Table 2.

The results of univariate and multivariate analyses of risk factors for 30-day mortality are shown in Table 3. The independent risk factors for mortality were relapsed and refractory underlying disease, use of corticosteroids, respiratory clinical source, septic shock, and meropenem-resistant GNB bacteremia. In contrast, the clinical response on day 7 was a protective factor for survival.

## 4. Discussion

This study evaluated the epidemiological, clinical, and outcomes characteristics of bacteremia episodes in patients with HMs and HSCT. In addition, risk-factors for 30-day mortality were identified. They included a large number of patients with neutropenia, high doses of corticosteroids, bacteremia with clinical sources, and MDR-GNB, with both ESBL and KPC production being the main mechanisms involved. GNB isolates presented a high rate of resistance to the antibiotics most frequently prescribed, which was higher in patients with HSCT. These findings are associated with a poor outcome, given that GNB resistant to meropenem was one of the risk-factors for 30-day mortality.

Several studies worldwide have focused on the epidemiology and outcomes of HM and HSCT patients with bacteremia, and on the risk-factors for 30-day mortality. However, there are few studies from Latin America.

In a multicenter study from the US, Zimmer A. et al. evaluated the first episodes of bacteremia in febrile neutropenic patients. Of 389 isolates, half were Gram-positive cocci, with viridans group streptococci being the most frequent (24%), and only 7% caused by Coagulase-negative staphylococci. Among GNB, susceptibility to cefepime, piperacillin–tazobactam, and carbapenems was 84%, 88%, and 96%, respectively. Thirty-day overall mortality rate was 9.6%. Unlike our study, there was a high proportion of Gram-positive cocci with different etiological profiles. In addition, a low mortality rate was observed, which was probably due to high susceptibility to the most frequent antibiotics used in neutropenic patients [28].

In a multicenter study carried out in Italy, Trecarichi et al. included 811 GNB episodes of bacteremia in patients with HM. The most frequent GNB isolates were *E. coli* (52.5%), *K. pneumoniae* (19.2%), and *P. aeruginosa* (14.6%). MDR-GNB represented 30.7%, and susceptibility to ceftazidime, piperacillin–tazobactam, and carbapenems was 61.9%, 66.5%, and 80.9%, respectively. The thirty-day mortality rate was 16.3%, being significantly higher in MDR isolates. Unlike our study, they found a higher proportion of *E. coli* and *P. aeruginosa* as etiological microorganisms. However, antimicrobial susceptibility and mortality were similar to our cohort [7].

Two multicenter studies from Latin America have been recently published. In one of them, Cruz-Vargas S. et al. from Colombia evaluated 195 episodes of bacteremia in solid tumors (55.8%) and HM, of which 32.4% were in neutropenic patients. GNB represented 68.9%, and resistance to third- and fourth-generation cephalosporins and carbapenems was evidenced in 22.4% and 10.9%, respectively. The resistance mechanisms in carbapenem-resistant isolates were mainly KPC production (87%). *E. coli* and *K. pneumoniae* were MDR in 17.4% and 44.4%, respectively. The thirty-day mortality rate was 25.6%. This cohort comprised GNB with resistance antimicrobial profile lower than ours. However, the reported mortality rate was higher and not related to MDR-GNB [9].

In the other paper, Rabagliati R. et al. published a multicenter study from the Andean region. Febrile neutropenia episodes in patients with acute leukemia or lymphoma were included. Bacteremia was detected in 161 of 416 episodes (38.7%), and GNB were isolated in 86%. Resistance to cefotaxime, piperacillin–tazobactam, cefepime, and carbapenems was 41.9%, 33.7%, 32%, and 22.7%, respectively, being higher in *K. pneumoniae* isolates. MDR *K. pneumoniae* and *E. coli* isolates were 61.7% and 12.5%. Regarding resistance mechanisms, 17.2% were ESBL producers and 11% carbapenemase producers, mainly KPC. The thirty-day mortality rate was 26.7%. Even though they found a much higher predominance of GNB as the cause of bacteremia, the rate of antimicrobial resistance patterns and resistance mechanisms in this study was similar to that reported in our HM patients; however, mortality rate was higher [6].

These are the major findings of the present study: First, patients with HSCT had a higher rate of MDROs than those with HM, with a large proportion of MDR-coagulase-negative staphylococci. In addition, bacteremia caused by this pathogen was more frequently observed in HSCT patients. These findings could be due to the central-venous catheter source and fluoroquinolone prophylaxis use, which were more frequently observed in this group. Second, *Klebsiella* spp. was the leading cause of GNB bacteremia in HSCT patients. Even though only the first episodes of bacteremia were included, most of them were nosocomially acquired, which could explain this relevant finding. Third, in terms of resistance pattern, GNB from the two cohorts had a high resistance rate to cefepime, piperacillin–tazobactam, meropenem, and amikacin, being higher in HSCT patients. Resistance to tigecycline and fosfomycin was also higher in this group, compared to HM patients. Moreover, the second most common resistance mechanism in GNB was KPC production, which was slightly higher in HSCT patients. Recent colonization with KPC-producing Enterobacterales and duration of hospitalization until bacteremia ≥ 10 days were more frequently reported in HSCT patients, which could explain the high resistance to meropenem in this cohort. Fourth, a low resistance rate to ceftolozane–tazobactam and ceftazidime–avibactam was observed in the isolates tested against these antibiotics. More importantly, those antibiotics have recently shown to improve outcomes in neutropenic patients with MDR-*P aeruginosa* and KPC-producing Enterobacterales [29,30]. Fifth, one of the most significant findings of the study is that GNB resistant to meropenem was a predictive factor for 30-day mortality. Therefore, identification of patients at risk for presenting these microorganisms could improve the outcome.

Recently, a clinical score was developed to stratify the risk for carbapenem-resistant Enterobacterales (CRE) bacteremia in cancer and HSCT patients. Three risk factors were identified: ≥10 days of hospitalization until bacteremia, previous antibiotic treatment > 7 days, and recent colonization with KPC-producing Enterobacterales. The score showed high specificity and adequate positive predictive value for having CRE bacteremia. Moreover, it revealed very low post-test probability of CRE occurrence in patients with none of the risk factors. It was internally validated with the bootstrap resampling technique and had good predictive performance [15]. Similarly, over the last few years, many studies focused on the use of machine learning to predict antibiotic resistance [31,32]. One of them could accurately predict the occurrence of carbapenem-resistant GNB in ICU patients [33]. In addition, Garcia-Vidal C. et al. developed a predictive model with machine learning algorithms for the detection of MDR GB bacteremia in neutropenic patients. The model had high specificity and negative predictive value [34]. Both clinical scores and machine learning algorithms could contribute to the appropriateness of empirical antibiotic treatments and a decrease in mortality.

Sixth, inappropriate antibiotic ET was not associated with 30-day mortality. In this large cohort, several risk factors for mortality were highly related to the severity of clinical presentation and underlying diseases, as reported in other studies [35,36]. In addition, those patients with refractory or relapse underlying disease, high doses of corticosteroids, respiratory sources of bacteremia, and septic shock who had a higher risk of death could benefit from combined AT, which in some studies is associated with lower mortality [37,38]. Seventh, even though 30-day mortality was high, the population had a large proportion of risk factors for mortality. In addition, the mortality rate was lower than that reported in other similar studies previously mentioned [6,9]. The seven-day clinical response was a protective factor for survival, probably related to the high rate of appropriate empirical treatment prescribed according to the patient’s clinical and epidemiological features. This supports the need to individualize the most suitable treatment approach for each patient.

Our study has some limitations that should be considered. First, we did not analyze neutropenic patients separately from the total cohort, which could be associated with a higher mortality rate during bacteremia episodes. However, we found that some studies showed no differences in overall mortality between neutropenic and non-neutropenic cancer patients [39]. Second, the study was conducted in a country with a high prevalence of infection caused by MDR-GNB. Therefore, our results may not be extrapolated to countries or centers with different epidemiology and antibiotic resistance patterns.

The strengths of our study rely on its prospective and multicenter design, which was carried out in healthcare facilities specialized in the treatment of patients with HM or HSCT, where a large number of bacteremia episodes were included. In addition, given that only the first bacteremia episodes were included, the results can more accurately represent this complex epidemiological scenario.

## 5. Conclusions

This study showed that GNB is the predominant cause of bacteremia in patients with HM or HSCT. They had an increased rate of MDR-GNB with a high level of resistance to the most frequent antibiotics used in this population, being higher in patients with HSCT. Resistance to meropenem was identified as one of the risk factors for 30-day mortality. In view of this finding, an individualized approach is crucial for the treatment of these patients. In addition, the identification of patients at risk of presenting carbapenem-resistant Enterobacterales bacteremia and their adequate treatment could improve their outcomes.

## Figures and Tables

**Table 1:** Baseline and epidemiological characteristics.

Variables	HM n = 920 n (%)	HSCT n = 357 n (%)	*p* *
Age (years) (median, IQR)	53 (37–64)	50 (37–59)	**0.002**
Male gender	508 (55.2)	235 (65.8)	**0.001**
Charlson comorbidity index ≥3	210 (22.8)	50 (14.1)	**<0.001**
Hematological diseases			
Acute leukemia	486 (52.8)	112 (31.4)	**<0.0001**
Lymphoma	278 (30.2)	117 (32.8)	0.37
Multiple myeloma	65 (7.1)	94 (26.3)	**<0.0001**
Myelodysplastic syndrome	47 (5.1)	26 (7.3)	0.13
CML/CLL	44 (4.8)	8 (2.2)	**0.03**
Stage of underlying cancer			
Recently diagnosed	379 (41.2)	11 (3.1)	**<0.0001**
Complete remission	124 (13.5)	209 (58.1)	**<0.0001**
Partial remission	85 (9.2)	57 (16)	**0.001**
Refractory	95 (10.3)	26 (7.3)	0.09
Relapse	237 (25.8)	55 (15.4)	**<0.0001**
Treatment of the underlying disease			
Chemotherapy (1 month prior to bacteremia)	663 (72.1)	227 (63.6)	**0.003**
Radiotherapy (1 month prior to bacteremia)	20 (2.2)	30 (8.4)	**<0.0001**
High dose of corticosteroids	316 (34.3)	133 (37.3)	0.32
Biological agents/anti-lymphocyte drugs	110 (12)	66 (18.5)	**0.002**
Recent hospitalization (1 month prior to bacteremia)	501 (54.5)	137 (3.4)	**<0.0001**
Neutropenia	638 (69.3)	286 (80.1)	**<0.0001**
High-risk neutropenia by their MASCC score	565 (88.5)	261 (91.2)	0.21
Neutropenia duration (days) (median, IQR)	14 (8–26)	13 (10–18)	0.48
Neutropenia > 10 days	406 (63.64)	211 (73.78)	**0.002**
Previous antibiotic use	437 (47.4)	168 (47.1)	0.88
Fluoroquinolone prophylaxis	113 (12.3)	95 (26.6)	**<0.0001**
Previous colonization by KPC-PE	59 (6.4)	39 (10.9)	0.05
Recent colonization by KPC-PE	53 (5.7)	33 (9.2)	**<0.001**
Duration of hospitalization until bacteremia (days) (median, IQR)	4 (0–14)	11 (5–15)	**<0.0001**

Abbreviation: HM: hematologic malignancies; HSCT: hematopoietic stem cell transplantation; IQR: interquartile range; CML: chronic myelogenous leukemia; CLL: chronic lymphocytic leukemia; MASCC: Multinational Association for Supportive Care in Cancer; KPC-PE: *Klebsiella pneumoniae* carbapenemase-producing Enterobacterales. * *p*-values obtained by chi-square for categorical variables and Mann–Whitney U-test for continuous variables. Bold: statistically significant.

**Table 2:** Clinical characteristics, antibiotic therapy, and outcome.

Variables	HM n = 920 n (%)	HSCT n = 357 n (%)	*p* *
Nosocomial bacteremia	616(66.9)	310 (86.9)	**<0.0001**
Healthcare-associated bacteremia	238 (25.9)	39 (10.9)	**<0.0001**
Community-acquired infection	66 (7.2)	8 (2.3)	**<0.0001**
Bacteremia with clinical source	641 (69.7)	275 (77)	**0.009**
Central venous catheter infection	277 (24.7)	131 (36.7)	**<0.0001**
Abdominal infection	149 (16.2)	65 (18.2)	0.38
Respiratory infection	92 (10)	26 (7.3)	0.13
Skin and soft tissue infection	83 (9)	15 (4.2)	**0.004**
Urinary tract infection	39 (4.2)	4 (1.1)	**0.005**
Severe mucositis	24 (2.6)	26 (7.3)	**<0.0001**
Perianal infection	22 (2.4)	10 (2.8)	0.67
Others	31 (3.7)	8 (2.4)	0.29
APACHE II score the day of bacteremia (median, IQR)	13 (10–17)	13 (9–16)	0.07
APACHE II score ≥ 20	143 (15.5)	44 (12.3)	0.14
Pitt score the day of bacteremia (median, IQR)	0 (0–1)	0 (0–2)	0.54
Pitt score ≥ 4	60 (6.5)	27 (7.5)	0.51
Empirical Antibiotic Therapy			
Piperacillin–tazobactam	378 (40.1)	155 (43.3)	0.44
Carbapenem	379 (41.1)	150 (42)	0.55
Vancomycin	321 (34.9)	150 (42)	**0.01**
Amikacin	109 (11.8)	61 (17.1)	**0.01**
Colistin	129 (14)	63 (17.6)	0.10
Cefepime	70 (7.6)	25 (7)	0.71
Appropriate EAT	775 (84.2)	298 (83.5)	0.73
Combined EAT	317 (34.5)	146 (40.9)	**0.03**
Definitive Antibiotic Therapy			
Piperacillin–tazobactam	198 (21.5)	81 (22.7)	0.65
Carbapenem	243(26.4)	100 (28)	0.56
Vancomycin	130 (14.1)	81 (22.7)	**<0.0001**
Amikacin	39 (4.2)	22 (6.2)	0.14
Colistin	82 (8.9)	45 (12.6)	**0.04**
Cefepime	66 (7.2)	25 (7)	0.91
Monotherapy DAT	798 (86.7)	298 (83.4)	0.13
Duration of DAT	12 (8–14)	11 (8–14)	0.13
Intensive care unit admission required	178 (19.3)	69 (19.3)	0.99
Septic shock development	166 (18)	61 (17.1)	0.68
Breakthrough bacteremia	62 (6.7)	32 (9)	0.17
7-day clinical response	740 (80.4)	294 (82.4)	0.43
7-day mortality	92 (10)	33 (9.2)	0.68
30-day mortality	161 (17.5)	63 (17.6)	0.95
Infection-related 30-day mortality	102 (11)	35 (9.8)	0.28

Abbreviation: HM: hematologic malignancies; HSCT: hematopoietic stem cell transplantation; IQR: interquartile range; EAT: empirical antibiotic therapy; DAT: definitive antibiotic therapy. * *p*-Values obtained by chi-square for categorical variables, and Mann–Whitney U-test for continuous variables. Bold: statistically significant.

**Table 3:** Risk factors for 30-day mortality.

Variable	Univariate Analysis			Multivariate Analysis		
	Non-Adjusted OR	95% CI	*p-*Value	Adjusted OR	95% CI	*p-*Value
Allogeneic HSCT	1.63	1.09–2.43	**0.016**	1.64	0.88–3.06	0.11
Relapse disease	1.53	1.11–2.11	**0.010**	1.69	1.04–2.73	**0.032**
Refractory disease	3.01	1.98–4.54	**<0.0001**	3.31	1.78–6.15	**<0.0001**
High-dose corticosteroids	2.17	1.62–2.91	**<0.0001**	2.13	1.38–3.30	**0.001**
Polymicrobial bacteremia	2.11	1.29–3.46	**0.003**	2.04	0.97–4.28	0.059
Breakthrough bacteremia	3.12	1.99–4.87	**<0.0001**	1.13	0.56–2.28	0.728
Meropenem-resistant GNB	4.98	3.48–7.12	**<0.0001**	1.99	1.05–3.77	**0.034**
Respiratory clinical source	2.96	1.97–4.46	**<0.0001**	2.27	1.23–4.16	**0.008**
Inappropriate EAT	1.67	1.17–2.39	**0.005**	1.04	0.56–1.92	0.888
Septic shock	19.72	13.88–28.01	**<0.0001**	8.29	5.15–13.22	**<0.0001**
APACHE II score ≥ 20	2.19	1.53–3.13	**<0.0001**	0.72	0.39–1.31	0.285
Pitt score ≥ 4	7.89	5.01–12.43	**<0.0001**	1.25	0.61–2.59	0.533
7-day clinical response	0.03	0.02–0.05	**<0.0001**	0.06	0.04–0.09	**<0.0001**

Multiple logistic regression model. Cox and Snell R^2^ = 0.353; Nagelkerke R^2^ = 0.584. Bold: statistically significant. Abbreviation: OR: Odds Ratio; 95% CI: 95% confidence interval; HSCT: hematopoietic stem cell transplantation; GNB: Gram-negative bacilli; EAT: empirical antibiotic treatment.

## Data Availability

Data are available upon request. Contact the corresponding author.
